# p53 Immunohistochemical Patterns in HPV-Independent Squamous Cell Carcinomas of the Vulva and the Associated Skin Lesions: A Study of 779 Cases

**DOI:** 10.3390/ijms21218091

**Published:** 2020-10-29

**Authors:** Natalia Rakislova, Laia Alemany, Omar Clavero, Adela Saco, Aureli Torné, Marta del Pino, Meritxell Munmany, Maria Teresa Rodrigo-Calvo, José Guerrero, Lorena Marimon, Naiara Vega, Beatriz Quirós, Belen Lloveras, Inmaculada Ribera-Cortada, Maria Alejo, Michael Pawlita, Wim Quint, Silvia de Sanjose, Jaume Ordi, VVAP Study Group

**Affiliations:** 1 Department of Pathology, ISGlobal, Hospital Clínic—Universitat de Barcelona, 08036 Barcelona, Spain; rakislova@clinic.cat (N.R.); masaco@clinic.cat (A.S.); MTRODRIGO@clinic.cat (M.T.R.-C.); JAGUERRERO@clinic.cat (J.G.); lorena.marimon@isglobal.org (L.M.); NVEGA@clinic.cat (N.V.); itribera@clinic.cat (I.R.-C.); 2 Unit of Infections and Cancer, Cancer Epidemiology Research Program, Catalan Institute of Oncology, IDIBELL, 08908 L’Hospitalet de Llobregat, Spain; alemanyvilchesico@gmail.com (L.A.); omarclavero@hotmail.com (O.C.); bquiros@iconcologia.net (B.Q.); 3 CIBER Epidemiologia y Salud Pública, 28029 Madrid, Spain; 4 Institute of Gynecology, Obstetrics and Neonatology, Hospital Clínic—Institut d´Investigacions Biomèdiques August Pi I Sunyer (IDIBAPS), Faculty of Medicine-University of Barcelona, 08036 Barcelona, Spain; ATORNE@clinic.cat (A.T.); mdelpino@clinic.cat (M.d.P.); mmunmany@clinic.cat (M.M.); 5 Department of Pathology, Hospital del Mar, 08003 Barcelona, Spain; blloveras@psmar.cat; 6 Department of Pathology, Hospital General d’Hospitalet, 08906 L’Hospitalet de Llobregat, Spain; maria.alejo@sanitatintegral.org; 7 Division of Molecular Diagnostics of Oncogenic Infections, Research Program Infection, Inflammation and Cancer, German Cancer Research Center (DKFZ), 69120 Heidelberg, Germany; m.pawlita@dkfz-heidelberg.de; 8 DDL Diagnostic Laboratory, 2288 Rijswijk, The Netherlands; wim.quint@ddl.nl; 9 National Cancer Institute (NCI), Rockville, MD 20850, USA; desanjose.silvia@gmail.com

**Keywords:** p53, *TP53*, vulvar squamous cell carcinoma, HPV-independent vulvar cancer

## Abstract

Human papillomavirus (HPV)-independent vulvar squamous cell carcinomas (VSCC) and its precursors frequently harbour *TP53* mutations. Recently, six p53 immunohistochemical (IHC) patterns have been defined, which have shown strong correlation with *TP53* mutation status. However, few studies have applied this new six-pattern framework and none of them exhaustively compared p53 IHC positivity and patterns between invasive VSCC and adjacent skin lesion. We performed p53 IHC in a series of 779 HPV-independent VSCC with adjacent skin and evaluated the IHC slides following the newly described classification. Some 74.1% invasive VSCC showed abnormal p53 IHC staining. A skin lesion was identified in 450 cases (57.8%), including 254 intraepithelial precursors and 196 inflammatory/reactive lesions. Two hundred and ten of 450 (47%) VSCC with associated skin lesions showed an abnormal p53 IHC stain, with an identical staining pattern between the VSCC and the adjacent skin lesion in 80% of the cases. A total of 144/450 (32%) VSCC showed wild-type p53 IHC both in the invasive VSCC and adjacent skin lesion. Finally, 96/450 (21%) VSCC showed p53 IHC abnormal staining in the invasive VSCC but a wild-type p53 staining in the skin lesion. Most of the discordant cases (70/96; 73%) showed adjacent inflammatory lesions. In conclusion, the p53 IHC staining and pattern are usually identical in the VSCC and the intraepithelial precursor.

## 1. Introduction

Vulvar squamous cell carcinomas (VSCC) may arise via human papillomavirus (HPV)-associated and -independent pathways [1,2]. Invasive VSCC are commonly preceded by an intraepithelial precursor [1,2], and each type of invasive VSCC, HPV-associated and -independent, has specific precursors with differential morphological and immunohistochemical (IHC) features. The precursor of HPV-associated VSCC, i.e., high-grade squamous intraepithelial lesion (HSIL), is a well-characterized lesion with clear-cut morphological features. In contrast, HPV-independent precursors are more challenging and poorly defined lesions. Several intraepithelial precursors have been described in association with HPV-independent VSCC. They include differentiated vulvar intraepithelial neoplasia (dVIN), the classical precursor of HPV-independent VSCC [3,4,5,6,7,8], but also other new entities that have recently been added to the list of HPV-independent premalignant lesions: HSIL-like lesion [9], also referred to as basaloid dVIN [10], vulvar acanthosis with altered differentiation (VAAD) [11], and the closely related differentiated exophytic vulvar intraepithelial lesion (DEVIL) [12]. Finally, chronic inflammatory skin lesions, such as lichen sclerosus (LS), and other inflammatory/reactive skin lesions, including lichen planus, lichen simplex chronicus, and other lesions, are frequently identified in these patients [13]. The morphologic features of dVIN are subtle [14] and not infrequently overlap with inflammatory lesions [13], vulvar HSIL [9], and VAAD/DEVIL [3].

*TP53* mutations are a common finding in HPV-independent VSCC and its classical precursor lesion, dVIN [5,15]. Indeed, recent molecular studies have shown that 40–70% of HPV-independent VSCC and dVIN harbor *TP53* mutations [3,4,5,6,7,8]. In contrast, VAAD and DEVIL are rarely associated with *TP53* abnormalities [3]. The most recent evidence has shown clear prognostic differences based on *TP53* mutated or wild-type status in VSCC [16]. p53 IHC has been used as an adjunct in the identification and classification of HPV-independent VSCC and its precursors [17]. However, the criteria of p53 IHC evaluation have remained poorly defined over the last decades. Recently, a molecularly based p53 IHC interpretation framework has been proposed [18,19], which has shown an excellent correlation with *TP53* mutational status [18] and high reproducibility among experienced gynecological pathologists [19]. Importantly, this new p53 IHC interpretation framework has the potential to define the prognostic relevance of *TP53* mutation status within HPV-independent VSCC and its precursor intraepithelial lesions.

Only two studies have applied this new six-pattern classification in VSCC and premalignant lesions [19,20], but in both of them the study populations were relatively small. In addition, none of the previous studies have analyzed simultaneously the p53 positivity/negativity and IHC staining pattern in the invasive VSCC and in the associated skin lesion. Thus, the objectives of this study are: (1) to examine the spectrum of p53 IHC patterns in a large series of well-characterized HPV-independent VSCC (HPV DNA-negative and p16-negative) with adjacent skin, and (2) to correlate the p53 IHC staining pattern in the invasive VSCC and in the adjacent skin lesions.

## 2. Results

Overall, 577/779 invasive VSCC (74.1%) showed an abnormal p53 IHC staining, and 202/779 (25.9%) showed wild-type p53 expression. Parabasal/diffuse (373 tumors, 47.8%), followed by basal overexpression (130 tumors, 16.7%) were the most frequent abnormal patterns observed. Null pattern was observed in 66 cases (11.4%), whereas cytoplasmic staining was identified only in eight VSCC (1.4%). The percentage of invasive VSCC with abnormal patterns of p53 IHC was significantly higher in cases arising in normal skin compared with VSCC with adjacent skin lesion (82.4% vs. 68.0%; *p* < 0.001).

Four hundred and fifty out of 779 cases (57.8%) showed at least one adjacent skin abnormality, and in 329 cases (42.2%) no skin lesions were identified. Intraepithelial precursors were identified in 254 cases (32.6%). dVIN was the most frequent intraepithelial precursor identified (186/779, 23.9%), followed by HSIL-like lesions (*n* = 46, 5.9%) and VAAD/DEVIL (*n* = 22, 2.8%). LS was identified in 36 cases (4.6%). Finally, other inflammatory/reactive lesions were recognized in 160 cases (20.5%). LS was identified as an accompanying lesion in 64 cases (25.2%) with intraepithelial lesions. An abnormal p53 IHC staining was identified in 129/186 (69.3%) of the dVIN, 34/46 (73.9%) of the HSIL-like lesions, 9/22 (40.9%) of VAAD/DEVIL, 9/36 (25.0%) LS, and 29/160 (18.1%) of inflammatory/reactive lesions.

**Figure 1:**
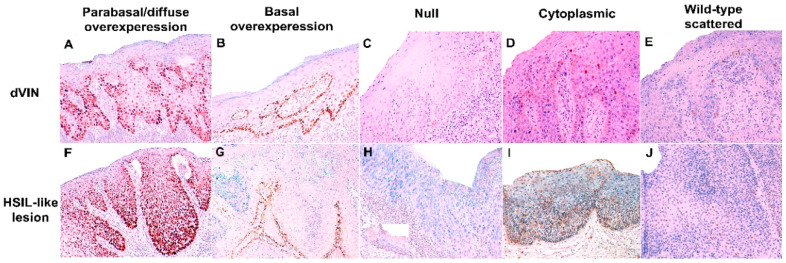
Examples of abnormal and wild-type p53 immunohistochemical expression in the two types of intraepithelial lesions. (**A**–**E**) Differentiated vulvar intraepithelial neoplasia (dVIN), and (**F**–**J**) high-grade squamous intraepithelial-like lesion (HSIL-like). Each column shows, respectively, the following pattern: First column, parabasal overexpression; second column, basal overexpression; third column, null; fourth column, cytoplasmic; fifth column, wild-type scattered pattern (p53 IHC immunostaining with hematoxylin counterstain, original magnification: 100×).

**Figure 2:**
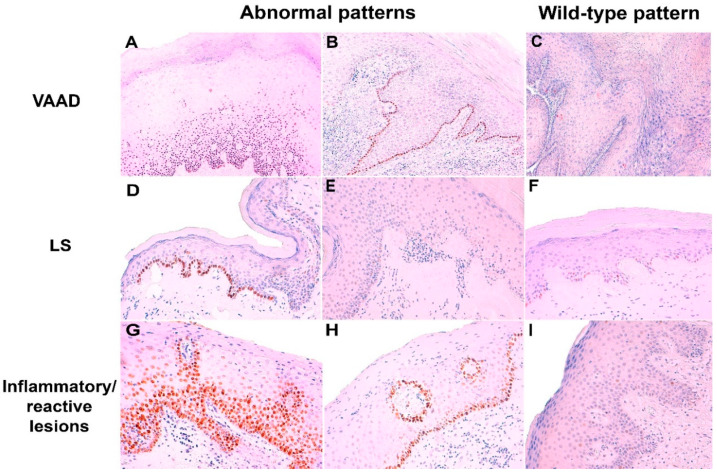
Examples of abnormal and wild-type p53 immunohistochemical expression in vulvar acanthosis with altered differentiation (VAAD), lichen sclerosus (LS) and inflammatory/reactive lesions. (**A**,**G**) Parabasal/diffuse patterns; (**B**,**D**,**H**) basal pattern; (**E**) null pattern (with wild-type staining in the hair follicle on the left); (**C**,**F**,**I**) wild-type scattered staining. (p53 IHC immunostaining with hematoxylin counterstain, original magnification: 100×).

Table 1 shows the p53 IHC patterns identified in the different intraepithelial precursors and inflammatory lesions. Parabasal/diffuse or basal overexpression were the most frequent p53 IHC pattern observed in dVIN and HSIL-like lesions, but 30.7% and 26.1% of these lesions, respectively, showed wild-type p53 expression. Wild-type scattered staining was the most frequent p53 IHC pattern in VAAD/DEVIL cases, but 40.9% of the cases showed basal or parabasal overexpression. Normal skin showed wild-type scattered pattern in all cases, although staining was frequently limited to only few cells. Representative examples of abnormal and wild-type p53 IHC patterns in the different intraepithelial and inflammatory lesions are shown in the Figure 1 and Figure 2.

**Figure 3:**
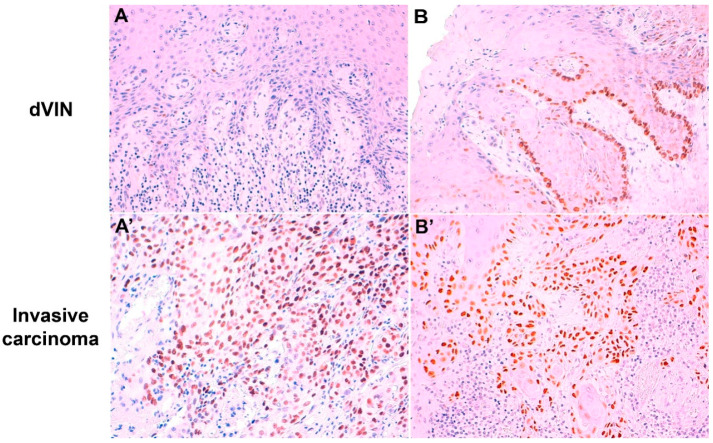
Two cases with discordant p53 immunohistochemical patterns between intraepithelial lesion and adjacent invasive carcinoma. (**A**): Differentiated vulvar intraepithelial neoplasia (dVIN) showing wild-type scattered p53 pattern; (**A’**) invasive carcinoma from the same case with p53 parabasal overexpression; (**B**): dVIN with p53 basal staining; (**B’**) invasive component from the same case showing p53 parabasal overexpression (p53 IHC immunostaining with hematoxylin counterstain, original magnification: 100× (**A**,**B**,**B’**) and 200× (**A’**)).

Table 2 shows correlation of the p53 IHC patterns between invasive VSCC and their respective adjacent skin lesions in 450 VSCC. Two hundred and ten out of 450 VSCC (46.6%) had abnormal p53 staining both in the adjacent skin lesion and in the invasive component. The type of an abnormal p53 IHC pattern in the invasive VSCC and in the skin lesion was identical in 80% of the cases (168/210). Discrepancies in the abnormal p53 IHC pattern were only observed between basal and parabasal/diffuse overexpression, whereas all invasive VSCC with null or cytoplasmic abnormal patterns had the same pattern in the surrounding skin lesion. One hundred and forty-four VSCC out of 450 (32.0%) had a wild-type p53 IHC both in the invasive tumor and adjacent skin lesion, with p53 pattern being coincident in all cases. Finally, 96 cases (21.3%) showed an abnormal p53 staining in the invasive VSCC and a discordant, wild-type p53 IHC expression in the adjacent skin lesion (16 dVIN, seven HSIL-like lesions, three VAAD/DEVIL, and 70 inflammatory lesions). Figure 3 shows two examples of discordant p53 IHC patterns between the intraepithelial lesion and the VSCC.

## 3. Discussion

Our study provides a representative picture of the distribution of the p53 IHC patterns in a large unselected sample of HPV-independent VSCC from a wide geographic origin. To our knowledge, only one study has applied the newly introduced six-pattern framework [19], predominantly in HPV-negative VSCC, but, unlike the present report, it did not provide the real distribution of the p53 IHC patterns due to pre-selection of cases. Moreover, the staining patterns of lesions with unusual morphology have not been previously explored. Other studies evaluating p53 IHC in VSCC and/or intraepithelial precursors were conducted before the six-pattern framework was introduced [21,22,23,24,25]. Thus, these studies did not recognize the newly introduced patterns, such as mid-epithelial or cytoplasm staining, and the basal pattern was not specifically recognized either, although at least some of them, were reported as abnormal [21,22,23,24]. Some of these studies only evaluated p53 IHC as positive or negative [6,22]. Therefore, comparisons with other studies might be biased in terms of pattern distribution. Indeed, whereas the overall frequency of p53 abnormal p53 IHC patterns in VSCC in our study (74.1%) is similar to the rates reported in the two recent studies that applied the six-pattern framework [18,19], they are higher than the percentages reported in previous studies that did not use this scheme [5,21,25,26]. Notably, the recent studies that have introduced [18] or applied [19] the new six-pattern framework, have shown a lower frequency of basal overexpression pattern (<5% and 12%, respectively) compared with our study (17%), whereas the rates of parabasal, null, and wild-type patterns are similar. The pre-selection of unusual patterns in the abovementioned studies might explain these differences. Remarkably, our study shows higher rates of p53 abnormalities in VSCC with no adjacent skin lesions. These differences might be casual and must be interpreted with caution as only one block was available for revision for each case. On the other hand, no series has specifically compared these two groups of cases. Further studies focused in confirming this finding are warranted.

Notably, the correlation of p53 IHC staining pattern in the invasive VSCC and in the synchronic adjacent skin lesion has been poorly evaluated in the previous literature. Recent evidence suggests that the abnormal p53 IHC staining in the skin lesion consistently matches with that of the adjacent invasive VSCC [3]. In keeping with these findings, in our study all invasive VSCC with wild-type p53 patterns had a wild-type p53 expression in the adjacent skin lesion. Moreover, when an abnormal pattern of p53 expression was observed in the VSCC the same pattern was identified in the skin lesion in 80% of the cases, with discrepancies only observed between basal and parabasal/diffuse overexpression. These basal/parabasal abnormal pattern’s variations are probably not relevant, as both basal and suprabasal patterns are consistently associated with *TP53* missense mutation [18]. On the other hand, 31.4% of the invasive VSCC with abnormal p53 expression and adjacent skin lesion, showed a wild-type p53 IHC expression in the skin lesion. However, most of these p53 wild-type skin lesions adjacent to mutant p53-VSCC were inflammatory/reactive lesions, thus, probably not clonally related to the tumor. It is noteworthy that dVIN or HSIL-like lesions with discordant staining pattern comprised only 5% of the total 450 cases. Indeed, a recent study has identified also a small proportion of dVIN that did not harbor *TP53* mutation in contrast with the invasive VSCC [3]. Interestingly, another study that identified a few p53 IHC wild-type dVIN lesions giving rise to a p53 IHC abnormal invasive VSCC, showed that the dVIN did harbor *TP53* mutations identical to those in the invasive tumor [18]. On the other hand, it has been hypothesized that p53 IHC overexpression might occur late in the progression of vulvar tumorigenesis [27].

In keeping with other series [18,19], mid-epithelial and cytoplasmic p53 IHC patterns were exceedingly rare in our study. Interestingly, only one lesion showed mid-epithelial pattern. This lesion was an HSIL-like lesion, previously described by our group [9,10]. Indeed, this type of staining is rare and has been recently noted in previous studies, mainly in HPV-associated VSCC and precursors [18,28,29]. Although it has been proposed that the mid-epithelial pattern is caused by senescence of HPV-infected tumor cells [29], it has also been rarely reported in HPV-independent VSCC [19]. On the other hand, the two cases with cytoplasmic staining were identified in VSCC with HSIL-like lesion and dVIN. Contrarily, other recent series only identified this pattern in a few VSCC without skin [19] or did not identify it in any of the cases [17].

Surprisingly, 40.9% of our VAAD/DEVIL lesions exhibited p53 IHC abnormal patterns of expression, mostly basal or parabasal pattern. These newly described entities, VAAD and DEVIL, are still controversial as little evidence is available [5,11,12]. In this regard, although the most recent study did not report any *TP53* mutations in VAAD or DEVIL [3,12,30], Nooij et al. identified *TP53* abnormality in one out of seven VAAD lesions [5].

Finally, similarly to other studies [17,31], we also identified a small proportion of LS and inflammatory/reactive lesions with p53 abnormal staining. However, accumulating evidence suggests that LS do not harbor *TP53* mutations [31,32] and p53 IHC abnormalities in these cases might be associated with ischemic stress [31]. Likewise, it is not uncommon for inflammatory/reactive lesions to show p53 alterations not related to mutation [17]. Lastly, although some authors have reported a complete absence of p53 staining in normal skin [33], we have identified scattered wild-type staining in all normal skin, although in some cases, it was limited to one to two cells.

One of the main strengths of the study is the inclusion of a large number of HPV-independent VSCC with cases collected from the five continents. Secondly, both histology and IHC analysis were performed in a centralized manner. There are also significant limitations in our study. The main weakness is that *TP53* mutational analysis was not performed. However, recent studies have shown the new p53 IHC interpretation framework has shown to be highly correlated with *TP53* mutational status, with 95% concordance in VSCC and 93% in intraepithelial lesions [18]. Secondly, only one paraffin block was available per case, and consequently, some cases considered as arising on normal skin could harbor an intraepithelial lesion in a different area, not represented in the study block. In addition, the fact that the cases were collected in different laboratories with variable fixation protocols might have affected in some degree the p53 staining distribution. Finally, the complete absence of any clinical and follow-up data prevents any prognostic correlation of the findings of this study.

In conclusion, our study provides a reliable picture of the prevalence of the different p53 IHC patterns in a large unselected sample of HPV-independent VSCC from a wide geographic origin and confirms the percentages provided in recent molecular-based studies. The study corroborates previous findings that the pattern of p53 IHC is usually similar in VSCC and the associated premalignant lesion. Finally, our series shows the need of additional studies addressing the *TP53* mutational profiles in VSCC and intraepithelial lesions with concordant and discrepant p53 IHC patterns and in cases with unusual morphological features.

## 4. Materials and Methods

### 4.1. Case Selection

A series of 1709 invasive VSCCs included in the VVAP study (international survey on HPV prevalence and type distribution in vulvar, vaginal, anal, and penile neoplasms) was reviewed. The series, which has been described in detail elsewhere [34], includes cases from 38 countries from the five continents (Mali, Mozambique, Nigeria, and Senegal in Africa; Argentina, Brazil, Chile, Colombia, Ecuador, Guatemala, Honduras, Mexico, Paraguay, Uruguay, the United States, and Venezuela in the Americas; Bangladesh, India, Israel, South Korea, Kuwait, Lebanon, Philippines, Taiwan, and Turkey in Asia; Austria, Belarus, Bosnia-Herzegovina, Czech Republic, France, Germany, Greece, Italy, Poland, Portugal, Spain, and the United Kingdom in Europe and Australia and New Zealand in Oceania). The study was approved by theCatalan Institute of OncologyEthics Committee (ref 91/07; date 10/05/2007).

All VSCC fulfilling the following inclusion criteria were included in the study: (1) Negative result for HPV detection by polymerase chain reaction (PCR); (2) negative result for p16 IHC in the invasive tumor; and (3) presence of at least 1 cm of skin surrounding or overlying the invasive tumor.

**Figure 4:**
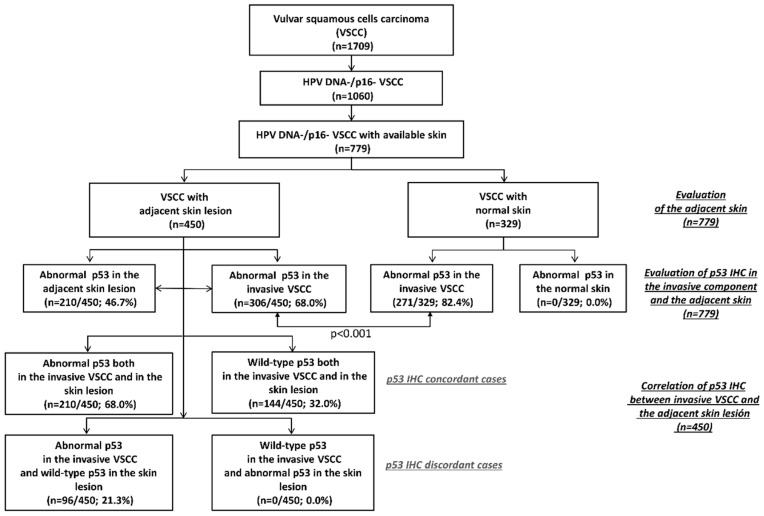
The study algorithm and results of histological and p53 immunohistochemical (IHC) evaluation of the adjacent skin and invasive vulvar squamous cell carcinoma (VSCC).

From 1709 VSCC included in the VVAP study, 1060 were HPV negative and p16 negative. Of them, 281 cases were excluded because no skin, or less than 1 cm of adjacent skin was available for revision. Overall, 779 cases fulfilled the inclusion criteria and were included in the analysis. The study algorithm is shown in Figure 4.

### 4.2. Tissue Preparation, Nucleic-Acid Isolation, and Human Papillomavirus (HPV) DNA Detection

DNA extraction was performed on whole sections of formalin-fixed paraffin-embedded tissue from surgical specimens or vulvar biopsies as previously described [35]. No microdissection was performed; in all cases, the analyzed tissue included the invasive tumor and the adjacent skin with the inflammatory and/or premalignant lesions, if present. Sectioning and sample preparation were carried out with the highest safety measures to avoid cross-contamination. Processing and pathology diagnosis were done by the ICO laboratory.

HPV DNA detection and typing were performed using SPF10 PCR, DEIA and the LiPA25 system (version 1, Labo Biomedical Products, Rijswijk, The Netherlands) as previously described [22]. Briefly, LiPA25 can be used to detect 25 high-risk and low-risk HPV types (6, 11, 16, 18, 31, 33, 34, 35, 39, 40, 42, 43, 44, 45, 51, 52, 53, 54, 56, 58, 59, 66, 68, 70, and 74). Each run contained negative and positive controls to monitor the efficiency of DNA isolation, PCR amplification, hybridization, and genotyping procedures.

### 4.3. Histological Evaluation

A single histological slide of each tumor was available for review. The squamous epithelium adjacent to the neoplasms was carefully evaluated in search of associated precursor lesions or inflammatory/reactive skin abnormalities. In order to establish the diagnosis of intraepithelial lesion, the lesion had the peripheral extension for at least 1 cm away from the invasive carcinoma to rule out possible peripheral intraepithelial extension of the invasive tumor [9,36].

Intraepithelial precursors included (1) dVIN, (2) HSIL-like lesions, and 3) VAAD/DEVIL. As inflammatory skin lesions we included LS as a separate category, but any other inflammatory/reactive skin conditions, including lichen simplex chronicus, lichen planus, and other non-specific inflammatory and/or reactive abnormalities, were grouped as a single category. The diagnosis of dVIN was based on the presence of atypical keratinocytes limited to the basal and parabasal layers in the context of a fully differentiated epithelium [14]. HSIL-like lesion was diagnosed based on the finding of small atypical cells throughout the epidermis, with a marked architectural disarray (basaloid subtype), by the presence of epidermis with wide and deep rete ridges, pleomorphism and easily recognizable koilocytic-like changes (warty subtype), or by the presence of combined basaloid and warty features (mixed basaloid/warty subtype) [9]. VAAD was diagnosed by absence of atypia and the presence of prominent acanthosis with variable verruciform architecture, loss of the granular cell layer with superficial epithelial cell pallor, and multilayered parakeratosis [11]; DEVIL’s diagnosis implied, in addition, focal preservation of granular layer and more striking parakeratosis [12]. VAAD and DEVIL lesions were grouped into a single category as VAAD/DEVIL. LS was diagnosed on the basis of homogenization of the collagen of the papillary dermis, alongside with other features described elsewhere [37]. The adjacent skin was considered normal in the absence of any intraepithelial precursor, LS, or any other premalignant or inflammatory/reactive skin lesions.

All 779 cases included in the VVAP study were evaluated by two pathologists (N.R. and J.O.). This evaluation was blind to the HPV detection and IHC results. The morphological evaluation included tumor subtype and characteristics of the adjacent skin with identification of inflammatory and/or intraepithelial precursors (if present). All discrepant results were reviewed in an adjudication meeting and a final diagnosis was established by consensus between the reviewers.

### 4.4. p53 Immunohistochemical (IHC)

IHC stainings were performed with the automated system TechMate 500 (Dako, Carpinteria, CA, USA), using the EnVision system (Dako). p53 was detected with the monoclonal antibody (clone DO-7; Dako, Carpinteria, CA, USA). Briefly, 4 μm sections were deparaffinized and hydrated through graded alcohols and water. Peroxidase was blocked for 7.5 min in ChemMate peroxidase-blocking solution (Dako). Then, the slides were incubated with the primary antibodies for 30 min and washed in ChemMate buffer solution (Dako). Then, the peroxidase-labeled polymer was applied for 30 min. After washing in ChemMate buffer solution, the glass slides were incubated with the diaminobenzidine substrate chromogen solution, washed in water, counterstained with hematoxylin, washed, dehydrated, and mounted. A positive control consisting of an ovarian serous papillary carcinoma was included in each section.

The staining was evaluated separately in the invasive tumor and in the adjacent skin. Figure 1 summarizes simplified flow of all the steps of the p53 IHC evaluation, and the stratification of the cases based on the histological findings. The evaluation was performed following recent p53 IHC pattern-based interpretation framework [18,19]. Briefly, p53 IHC staining was classified into six major categories: two normal (wild-type) and four abnormal (mutant) patterns. Wild-type patterns included: (1) scattered and (2) mid-epithelial (basal sparing) staining. Scattered pattern was defined as heterogeneous, dispersed nuclear staining in the basal and/or parabasal layer. Mid-epithelial pattern was diagnosed when there was moderate to strong nuclear p53 staining in the parabasal layers staining, with notable basal sparing. Abnormal (mutant) p53 staining patterns were classified into (1) basal overexpression, (2) parabasal (diffuse) overexpression, (3) null, and (4) cytoplasmic patterns. Basal pattern was defined as continuous, strong nuclear staining of basal layer. The same basal staining but with suprabasal extension was classified as p53 parabasal (diffuse). The p53 null pattern was characterized by a complete absence of staining in the tumor and/or intraepithelial lesion. Finally, diffuse cytoplasmic staining with or without nuclear expression was classified as a cytoplasmic pattern. The null and cytoplasmic patterns required an intrinsic positive control (non-lesional skin, stromal, or inflammatory cells) [8].

## Figures and Tables

**Table 1:** Immunohistochemical expression of p53 in the different intraepithelial lesions adjacent to vulvar squamous cell carcinoma (VSCC).

	Pattern of p53 Expression					
	Abnormal Patterns				Wild-Type Patterns	
Adjacent Skin Lesion	Basal Overexpression	Parabasal/Diffuse Overexpression	Null	Cytoplasmic	Scattered	Mid-Epithelial
**Intraepithelial precursors**						
dVIN (*n* = 186)	48 (25.8%)	67 (36.0%)	13 (7.0%)	1 (0.5%)	57 (30.7%)	0 (0%)
HSIL-like lesion (*n* = 46)	2 (4.3%)	27 (58.7%)	4 (8.7%)	1 (2.2%)	11 (23.9%)	1 (2.2%)
VAAD/DEVIL (*n* = 22)	5 (22.7%)	4 (18.2%)	0 (0%)	0 (0%)	13 (59.1%)	0 (0%)
**Inflammatory lesions**						
Lichen sclerosus (*n* = 36)	6 (16.7%)	0 0%)	3 (8.3%)	0 (0%)	27 (75.0%)	0 (0%)
Other inflammatory/reactive lesions (*n* = 160)	14 (8.7%)	15 (9.4%)	0 (0%)	0 (0%)	131 (81.9%)	0 (0%)
Total	75	113	20	2	239	1

dVIN: differentiated vulvar intraepithelial neoplasia; HSIL-like: high-grade squamous intraepithelial lesion-like; VAAD/DEVIL: vulvar acanthosis with altered differentiation/differentiated exophytic vulvar intraepithelial lesion.

**Table 2:** Correlation of the p53 immunohistochemical patterns between the invasive squamous cell carcinoma of the vulva and the adjacent skin lesions in the 450 vulvar squamous cell carcinoma (VSCC) with abnormal skin. The cases in which the pattern of p53 expression in the invasive carcinoma and in the associated skin lesion is coincident are in bold and highlighted in grey (diagonal of coincidence).

	Pattern of p53 Expression in the Adjacent Skin Lesion					
	Abnormal Patterns (*n* = 210)				Wild-Type Patterns (*n* = 240)	
Pattern of p53 Expression in the Invasive Carcinoma	Basal Overexpression	Parabasal/Diffuse Overexpression	Null	Cytoplasmic	Scattered	Mid-Epithelial
**Abnormal patterns (*n* = 306)**						
Basal overexpression (*n* = 84)	**39 (46.4%)**	6 (7.2%)	0 (0%)	0 (0%)	39 (46.4%)	0 (0%)
Parabasal/Diffuse overexpression (*n* = 193)	36 (18.7%)	**107 (55.4%)**	0 (0%)	0 (0%)	50 (25.9%)	0 (0%)
Null (*n* = 27)	0 (0%)	0 (0%)	**20 (74.1%)**	0 (0%)	7 (25.9%)	0 (0%)
Cytoplasmic (*n* = 2)	0 (0%)	0 (0%)	0 (0%)	**2 (100%)**	0 (0%)	0 (0%)
**Wild-type patterns (*n* = 144)**						
Scattered (*n* = 143)	0 (0%)	0 (0%)	0 (0%)	0 (0%)	**143 (100%)**	0 (0%)
Mid-epithelial (*n* = 1)	0 (0%)	0 (0%)	0 (0%)	0 (0%)	0 (0%)	**1 (100%)**
Total	75	113	20	2	239	1
